# Resolution of Disseminated Intravascular Coagulation in a Patient with COVID-19 and Associated Sepsis—Induced Neutropenia

**DOI:** 10.3390/medicina57020106

**Published:** 2021-01-24

**Authors:** Pierpaolo Di Micco, Michele Imparato, Giuseppe Lubrano, Donatella Iannuzzo, Luca Fontanella, Lucio Improta, Maria Rita Poggiano, Ciro Salzano, Alessio Rodolico, Andrea Fontanella

**Affiliations:** 1 Department of Medicine, Ospedale Buonconsiglio Fatebenefratelli di Napoli, 80122 Naples, Italy; micheleimparato@gmail.com (M.I.); donatella.iannuzzo@gmail.com (D.I.); lucafontanella1@gmail.com (L.F.); marypoggiano@gmail.com (M.R.P.); cirosalzano89@gmail.com (C.S.); a.rodolico14@gmail.com (A.R.); andreafontanella52@gmail.com (A.F.); 2 Intensive Care Unit, Ospedale Buonconsiglio Fatebenefratelli di Napoli, 80129 Naples, Italy; lubrano.giuseppe@fbfna.it; 3 Laboratory Medicine, Ospedale Buonconsiglio Fatebenefratelli di Napoli, 80129 Naples, Italy; improta.lucio@fbfna.it

**Keywords:** COVID-19, DIC, sepsis, neutropenia, lymphocytopenia

## Abstract

COVID-19 has been associated with a hypercoagulable state and thrombotic events. Venous thromboembolism has been the most commonly reported type of thrombosis but also arterial thrombosis and disseminated intravascular coagulation in inpatients have been described frequently in several clinical experiences. Patients with COVID-19, because of its tendency to induce leucopenia and overlapping of bacterial infection, may experience sudden disseminated intravascular coagulation (DIC), as in the case that we report here. However, early diagnosis and treatment may be associated with positive resolution of these severe complications.

## 1. Introduction

Coagulopathy with a trend towards a hypercoagulable state and thrombotic complications has been described since the first reports on COVID-19 were presented [1]. Disseminated intravascular coagulation (DIC) is an induced complication associated with several infections and it has been reported also as a complication in patients with COVID-19 [2]. DIC, in fact, occurs when the viral damage is associated with endothelial damage that may also induce ischemia of tissues and organs [3]. However, DIC is also a well-known complication of bacterial sepsis [4,5] that may be a consequence of viral infections and/or of immunodeficiencies such as lymphocytopenia or neutropenia [6,7,8]. SARS-CoV-2 is one virus which is able to induce lymphocytopenia [9], while neutropenia has been rarely reported [10].

We here report a case of a 63-year-old male who, during hospitalization for COVID-19, developed sudden neutropenia that induced bacterial sepsis and associated DIC.

## 2. Case History

LR, a 63-year-old male, required hospitalization for bilateral ground glass pneumonia due to SARS-CoV-2 infection (i.e., COVID-19). In the emergency room, he presented with fever and dyspnea, and a nasopharyngeal swab revealed positivity for SARS-CoV-2 infection; his laboratory values were typical of COVID-19, with an increase in C reactive protein (CRP), lactate deydrogenase (LDH), d-dimer, fibrinogen and with a reduced count of lymphocytes (Table 1). His treatment was based on full doses of systemic antibiotics (i.e., piperacilline-tazobactam 13.5 g daily; full doses of dexamethasone (i.e., 6 mg daily), prophylactic doses of enoxaparin (i.e., 4000 U daily), systemic intravenous fluconazole (i.e., 100 mg daily), oxygen therapeutic support with high-flow nasal cannula (HFNC)) [11]. The patient underwent this treatment without side effects for 10 days, with an improvement in his overall respiratory performance, improvement in pulse oximetry and regression of fever; his laboratory markers also revealed an improvement, as reported in Table 1, and Doppler ultrasound scan excluded deep vein thrombosis of the lower limbs.

**Figure 1:**
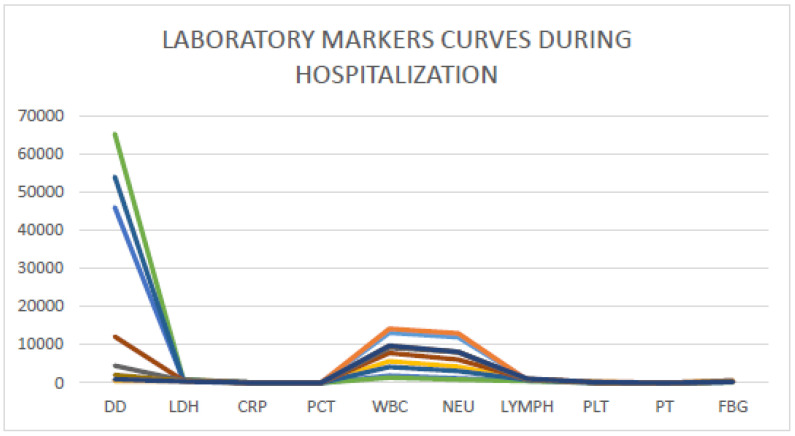
Laboratory markers curves during the hospitalization of reported patients. DD: d-dimer; LDH: lactate dehydrogenase; CRP: C reactive protein; PCT: procalcitonin; WBC: white blood cells; NEU: neutrophils; LYMPH: lymphocytes; PLT: platelets; PT: prothrombin time; FBG: fibrinogen.

Starting from day 11, the patient experienced chills twice daily, with new occurrence of fever, and new laboratory screening revealed sudden neutropenia with associated increase in d-dimer (i.e., 56,000 mcg/dL) and decrease in platelets (i.e., 46.000 mmcube). CRP showed a new strong increase and also procalcitonin showed a relevant and pathological increase. Assessment of clinical signs and symptoms was repeated on the following day and laboratory data confirmed the previously observed trend (Table 1) (Figure 1). Furthermore, several blood cultures were performed while the patient was experiencing chills in order to detect bacterial sepsis but none of them identified a bacterial pathogen.

We implemented a withdrawal of enoxaparin and desamethazone and we began pulse therapy with intravenous 3 g methylprednisolone on the first day, 2 g on the second day and 1 g on the third, with an associated a single shot of subcutaneous growth colony factor lenograstrin (i.e., 34 Millions UI/mL daily) in order to improve the count of neutrophils. We also started treatment of DIC with 2 units twice daily of fresh frozen plasma for three consecutive days in order to restore induced coagulopathy and prevent microthromboses of tissues and organs.

From the first day of treatment, the patient showed a clinical improvements concerning chills and fever and progressive improvements were recorded in laboratory markers, as reported in Table 1 and Figure 1.

After three days of the new therapeutic approach, all treatments were interrupted (also antibiotics and antimycotics) and clinical surveillance was performed for two additional days; then, the patient was dismissed, with improved clinical condition with normal laboratory markers and with improved lung performance (Table 1) on day 17 of his hospitalization.

## 3. Discussion

DIC is always a consequence of another underlying disease or infection [12]. Bacterial sepsis is more frequently associated with DIC because the bacterial wall is able to induce hyperactivation of the clotting system by several mechanisms [4,5,12]; however, hyperactivation of the clotting system with a trend toward DIC has been described during viral infections of COVID-19 since the first reports were presented from China [1,2]. SARS- CoV-2 is able to induce a hypercoagulable state via cytokine storm [13] and the induction of the peptidase cascade as angiotensin-converting enzyme (ACE) as complement toward the kallikrein system, which may activate clotting factor XII and so the intrinsic pathway of coagulation [14,15,16]. The hyperaction of the peptidases and clotting system, present also in other forms of DIC, induces an increase in d-dimer and the progressive consumption of clotting factors until the decrease in fibrinogen and platelets results in multiple bleedings and shock.

On the other hand, SARS-CoV-2 may also induce alteration of white blood cells and lymphocytopenia has been frequently reported in the first phases of infection. However, in severe clinical forms of COVID-19, neutropenia is also described and neutropenia is frequent in febrile patients with bacterial sepsis for other reasons.

In the case that we reported, the occurrence of neutropenia was quickly followed by the clinical complications of DIC and systemic sepsis. The early identification of both complications permitted us to begin treatment rapidly for neutropenia and DIC with early restoration of all clinical and laboratory parameters including neutrophils, platelets and procalcitonin.

The management of this case may contribute to the improved clinical screening of patients with COVID-19 and associated DIC, because early identification and treatment could allow good outcomes following these severe complications.

## Figures and Tables

**Table 1:** Laboratory test results of described patient.

Laboratory Test	Day 1	Day 3	Day 7	Day 10	Day 11	Day 12	Day 13	Day 14	Day 15	Day 16	Day 17
LDH (U/L)	765	802	631	501	690	860	712	614	603	501	389
CRP (mg/L)	123	147	96	49	85	114	91	62	39	9	6
Procalcitonin (ng/mL)	0.31	0.26	0.19	0.16	1.5	2.3	1.9	1.1	0.8	0.6	0.2
WBC (mmcube)	13,150	14,200	9180	5691	1900	1400	4203	7802	9201	9650	9730
Neutrophils	11,980	13,000	8120	4260	1215	890	3120	6123	8123	8003	8115
Lymphocytes	900	850	750	906	650	500	790	990	900	1100	1110
Platelets	206	401	352	263	63	46	79	112	115	126	169
Prothrombin time (INR)	1.08	1.02	1.03	1.06	1.42	1.75	1.53	1.3	1.1	1.06	1.09
Fibrinogen (mg/dL)	612	602	630	590	260	202	301	402	289	302	236
D-dimer (ng/dL)	650	712	502	509	46,000	65,023	54,000	12,361	4526	2103	960

LDH: Lactate dehydrogenase; CRP: C reactive protein; WBC: white blood cells.

## Data Availability

Data sharing not applicable.
